# High-Dose Methotrexate for the Treatment of Relapsed Central Nervous System Erdheim-Chester Disease

**DOI:** 10.1155/2014/269359

**Published:** 2014-06-16

**Authors:** Prahlad Ho, Carole Smith

**Affiliations:** Department of Clinical Haematology, Austin Health, Heidelberg, VIC, Australia

## Abstract

Erdheim-Chester disease (ECD) is a rare multisystem non-Langerhans histiocytosis. CNS involvement is a major complication, which is often rapidly progressive and confers a poor prognosis. However, treatment of CNS ECD is difficult due to poor CNS penetrance by the most effective chemotherapeutic drugs commonly used in this disorder (e.g., interferon and cladribine). We describe a case of a 60-year-old lady with a 5-year history of stable systemic ECD who presented with new brainstem lesions and rapid, steroid-refractory neurological deterioration which required immediate intervention. High-dose methotrexate was chosen due to its rapid onset of action and excellent CNS penetration. Her neurological deterioration was quickly arrested with significant functional improvement, which was sustained for 4 months with consolidation doses of high-dose methotrexate. Subsequent treatment with cladribine and interferon did not confer any appreciable clinical improvement. High-dose methotrexate is effective in controlling rapidly progressive CNS ECD and should be considered as a salvage agent prior to commencement of more definitive treatment.

## 1. Introduction

Erdheim-Chester disease (ECD) is a rare non-Langerhans histiocytosis characterised by multisystem involvement including skeletal, skin, retroperitoneal, retrobulbar, cardiac, and central nervous system (CNS) disease [1]. Cardiac and CNS complications typically confer worse prognosis and rapid progression [2, 3]. Histologically, the disease is characterised by foamy lipid-containing histiocytes, touton-like giant cells, which are CD1a negative and S100 negative on immunohistochemistry. Recent studies have also demonstrated that approximately half of the cases express BRAF V600E mutation [4], indicating the clonality of the disease in these cases. ECD was previously thought to be benign. The clinical course of this condition is varied, ranging from asymptomatic disease requiring no intervention to a rapidly progressive disease, which is often resistant to treatment. CNS involvement is common, affecting half of ECD cases [5, 6]. Typically, CNS disease occurs in the setting of wide spread systemic disease and confers a poor prognosis.

There remains no definitive treatment for ECD, though various agents like cladribine, cyclosporine, steroids, and radiotherapy have been trialled with variable success [3]. A recent multicentre retrospective analysis [3] has demonstrated that interferon-alpha may be useful for non-CNS systemic disease, particularly if it is given for more than 3 months. However, the efficacy of interferon-alpha in CNS disease remains uncertain, particularly since it has poor CNS penetrance (CSF: plasma ratio of 0.033 after IV administration) and has a slow onset of action [7]. Similarly, recently a case series reported excellent responses to treatment with vemurafenib in patients who possess the BRAF V600E mutation [8], but unfortunately vemurafenib also does not cross the blood-brain barrier [9].

Hence, despite recent diagnostic and therapeutic advances, there remains no effective treatment for rapidly progressive CNS Erdheim-Chester disease.

## 2. Case Report

**Figure 1:**
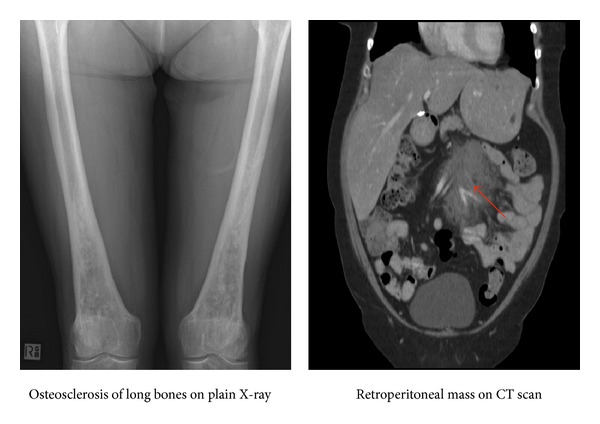
Radiological evidence of systemic features of Erdheim-Chester disease in our patient.

**Figure 2:**
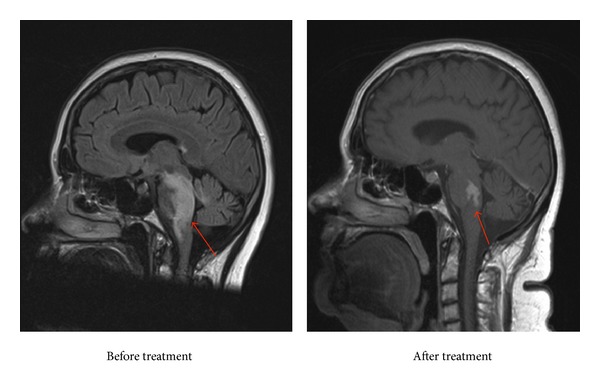
MRI brain demonstrating brainstem and cerebellar lesions before and after 2 cycles of with high-dose methotrexate.

We describe a case of a 60-year-old lady with a five-year history of stable non-CNS ECD characterised by radiological evidence of retrobulbar involvement, osteosclerosis of the long bones, and retroperitoneal fibrosis leading to renal artery stenosis and resistant hypertension (Figure 1). Her disease had remained stable for 3.5 years after commencing azathioprine and tamoxifen. She presented with 4 days of progressive diplopia and right arm numbness. Neurological examination revealed a horizontal gaze palsy, right arm paraesthesia, and mild weakness (4/5). Magnetic resonance imaging (MRI) of her brain revealed an extensive lesion involving her brainstem and cerebellum (Figure 2) but whole body positron emission tomography (PET) scan revealed stable systemic disease outside the CNS. Cerebrospinal fluid (CSF) analysis showed raised protein (3.12 g/L) but no evidence of infection or malignancy. Due to the position of the lesion, a brain biopsy was not performed as there was high risk of permanent neurological damage. A presumptive diagnosis of CNS relapse of ECD was made due to her long history of ECD and the unlikeliness of other possible diagnoses.

During the first 72-hour period and despite the steroid therapy, our patient rapidly developed dysarthria, progressive gaze palsy, and worsening ataxia and weakness, rending her bed-bound. This necessitated urgent treatment. We chose high-dose methotrexate (8 g/m^2^) due to its excellent CNS penetration and known therapeutic effect on CNS lymphoid malignancies. Interferon-alpha was not ideal due to its slow onset of action and poor CNS penetration. Vemurafenib was not available at that time. This treatment arrested the rapid progression and led to resolution of dysarthria and significant improvement in her ataxia and gaze palsy. A postinduction MRI brain showed a reduction in the size of her brainstem and cerebellar lesions and her CSF protein reduced to 0.53 g/L (Figure 2).

She maintained her neurological recovery with consolidation doses of high-dose methotrexate (3.5 g/m^2^) for 4 months. However, she subsequently relapsed and did not respond to interferon-alpha and subsequently cladribine, both of which did not produce a clinical or radiological improvement. She subsequently died due to progressive neurological disease, approximately 14 months after initial diagnosis of CNS ECD. An autopsy was performed and the brain lesion in the cerebellum and brain stem demonstrated numerous degenerative histiocytes, with an immunophenotype of CD68+/S100−/CD1a−/CD45+, consistent with treated ECD. The BRAF V600E mutation on autopsy was negative.

## 3. Discussion

The ability to treat CNS ECD relies on the ability of chemotherapeutic agents crossing the blood-brain barrier. High-dose methotrexate appears to be an effective agent in controlling the rapidly progressive effects of CNS ECD, due to its excellent CNS penetration and rapid effect. However, as described in this case report, methotrexate alone is not sufficient in controlling the overall nature of this disease and only has a temporary and palliative effect. Hence, further consolidation therapy, or even combination therapy, should be considered in order to have a more sustained disease control. Interferon-alpha and vemurafenib may have a role in consolidative therapy, particularly given their track record and effect on systemic disease, but is limited by CNS penetration.

## 4. Conclusion

High-dose methotrexate may be an effective salvage agent in patients with CNS ECD and should be considered in patients with rapidly progressing neurological disease prior to the commencement of a more definitive systemic agent.
